# Conceptualizing Treatment Strategies for Diabetic Kidney Disease: the Importance of Early Diagnosis and Treatment

**DOI:** 10.14789/ejmj.JMJ24-0031-P

**Published:** 2024-12-31

**Authors:** TOMOHITO GOHDA, SHINJI HAGIWARA, KENICHIRO ABE, HITOMI HIROSE, KENTA SHIMOZAWA, CHIAKI KISHIDA, HIROKO SAKUMA, ERI ADACHI, TAKEO KOSHIDA, YUSUKE SUZUKI, MAKI MURAKOSHI

**Affiliations:** 1 Department of Nephrology, Juntendo University Faculty of Medicine, Tokyo, Japan; 1 Department of Nephrology, Juntendo University Faculty of Medicine, Tokyo, Japan

**Keywords:** diabetic nephropathy, diabetic kidney disease, chronic kidney disease, albuminuria, treatment

## Abstract

Chronic kidney disease (CKD) attributed to diabetes, termed diabetic kidney disease (DKD), is increasing with the rising global prevalence of diabetes. Patterns of DKD onset and progression have shifted in recent years because of population aging and advances in the treatment of diabetes. Prevention of the onset and progression of micro/macro-albuminuria is possible through comprehensive and strict management of lifestyle, blood glucose, blood pressure, and lipids in people with diabetes and early DKD. Renin-angiotensin system (RAS) inhibitors have also been shown to effectively slow the progression of CKD in people with diabetes and micro/macro-albuminuria. However, the effect of improving kidney outcomes with RAS inhibitors in people with advanced DKD is limited, and the residual risk remains very high. A recent rapid expansion of treatment options include sodium-glucose co-transporter-2 inhibitors, non-steroidal mineralocorticoid receptor antagonists, and glucagon-like peptide-1 receptor agonists, which have demonstrated additional protective effects for the kidneys when used in addition to the standard therapy with RAS inhibitors, even in people with advanced DKD. Early diagnosis and therapeutic intervention can be expected to delay progression to end-stage kidney failure. This perspective outlines the diagnostic and therapeutic evolution of DKD to date.

## Introduction

There are approximately 20 million people with chronic kidney disease (CKD) in Japan, meaning that one in five adults is affected. This prevalence highlights CKD as an emerging national health concern. The main causes of CKD are lifestyle- related diseases such as diabetes and hypertension. Diabetic kidney disease (DKD) has become the most common disease requiring renal replacement therapy (RRT), currently accounting for about 40% of cases^1)^. In recent years, the proportion of transitions from DKD to RRT has decreased slightly because of advances in diabetes treatment. Meanwhile, the proportion of nephrosclerosis, thought to be caused by hypertension and arteriosclerosis, has gradually increased^2)^, overtaking chronic glomerulonephritis as the second most common cause of RRT.

For chronic glomerulonephritis, early diagnosis through health screenings and treatment advancements with immunosuppressants and biologics have decreased the rate of progression to end-stage kidney disease (ESKD). Previously, there were no treatments other than renin-angiotensin system (RAS) inhibitors that proved to suppress progression to ESKD or doubling of serum creatinine in people with diabetes and macroalbuminuria^3, 4)^. However, new treatments such as sodium-glucose co-transporter-2 (SGLT2) inhibitors, non-steroidal mineralocorticoid receptor antagonists (nsMRAs), and glucagon-like peptide-1 (GLP-1) receptor agonists (GLP-1RA) have been shown to effectively slow CKD progression^5-7)^. In this article, we discuss the factors influencing the combinations and sequence of these new treatments in people with heterogeneous DKD.

## Diversification of the onset and progression of DKD

**Figure 1:**
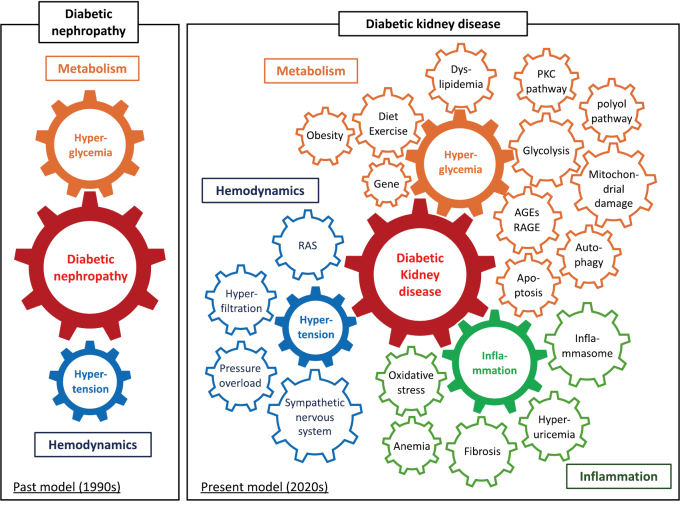
Pathogenesis of diabetic nephropathy and diabetic kidney disease Thirty years ago, diabetic nephropathy was understood as being driven by hyperglycemia and hypertension. We now understand that diabetic kidney disease involves various causes to different extents, leading to significant individual differences in onset and progression patterns. Abbreviations: AGEs, advanced glycation end products; PKC, protein kinase C; RAGE, receptor for AGEs; RAS, renin-angiotensin system

**Figure 2:**
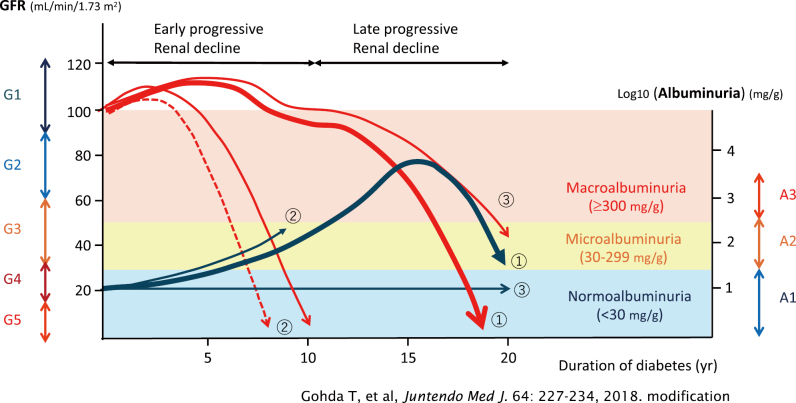
Diversified clinical course in people with diabetes and chronic kidney disease The clinical course of people with diabetes and chronic kidney disease can be classified into several types. (1) GFR decline starts after the onset of macroalbuminuria (classical diabetic kidney disease), (2) GFR decline precedes the onset of macroalbuminuria (early decliner), (3) GFR declines without increasing micro/macroalbuminuria (atypical diabetic kidney disease). Abbreviations: GFR, glomerular filtration rate

**Figure 3:**
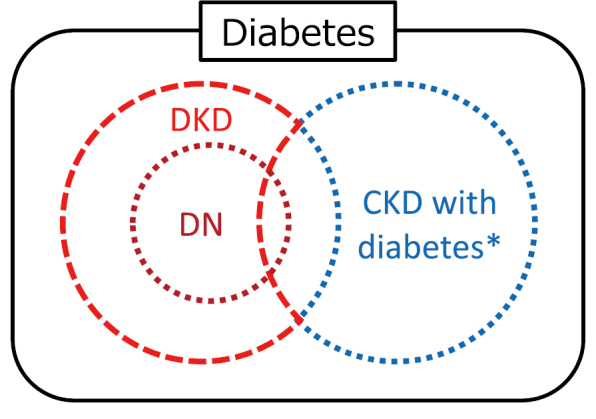
Concept of diabetic nephropathy, diabetic kidney disease, and chronic kidney disease with diabetes Diabetic kidney disease is a concept that encompasses diabetic nephropathy. Accurately distinguishing between diabetic kidney disease and diabetic nephropathy or chronic kidney disease with diabetes is often difficult and may require a kidney biopsy. *CKD with diabetes includes nephrosclerosis, obesity-related nephropathy, focal segmental glomerulosclerosis, IgA nephropathy, membranous nephropathy, membranoproliferative glomerulonephritis, polycystic kidney disease, Fabry disease, interstitial nephritis, lupus nephritis, and so on. Abbreviations: CKD, chronic kidney disease; DKD, diabetic kidney disease; DN, diabetic nephropathy

Thirty years ago, diabetic nephropathy was understood as being driven primarily by hyperglycemia, with hypertension being a secondary factor. However, the understanding of the pathophysiology of diabetic nephropathy has progressed over time, revealing that a wide range of factors are involved during onset and progression (Figure 1)^8)^. Pathological findings are useful in diabetic nephropathy, but clinical diagnosis is based on a typical progression pattern. After the onset of diabetes, persistent hyperglycemia usually leads to a gradual increase in urinary albumin levels from normal to microalbuminuria and then to macroalbuminuria, followed by a steep decline in glomerular filtration rate (GFR), finally leading to ESKD. Recently, however, there has been an increase in atypical cases where kidney function declines to ESKD without macroalbuminuria (Figure 2)^9)^. Therefore, there is an increasing number of people with diabetes who do not fall under the previously understood framework of classical diabetic nephropathy. This has led to the proposal of the inclusive concept of DKD (Figure 3).

Clinically, DKD is diagnosed when there are no clear signs of kidney disease caused by factors other than diabetes, combined with increased urinary albumin excretion (>30 mg/g) and/or decreased GFR (<60 mL/min/1.73 m^2^). However, distinguishing between DKD and nephrosclerosis can be difficult without a kidney biopsy, as people with diabetes often have comorbidities such as obesity, hypertension, and dyslipidemia. Pathologically, even if DKD is present, the relative contributions from diabetes and hypertension (arteriosclerosis) significantly affects the pattern of onset and progression.

## Early diagnosis and treatment

**Figure 4:**
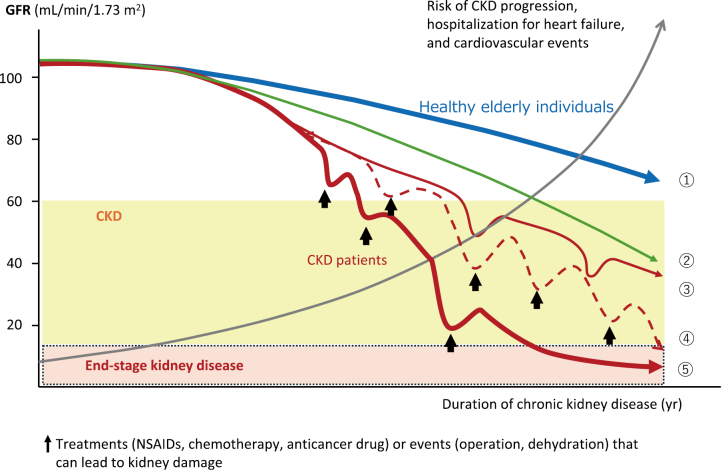
Trajectories in kidney function decline in healthy individuals and people with chronic kidney disease The clinical course of healthy individuals and people with CKD can be classified into several types. (1) Healthy individuals in which GFR declines with aging but remains above 60 mL/min/1.73 m^2^ throughout life (No CKD), (2) Individuals in which GFR declines with aging, resulting in a GFR below 60 mL/min/1.73 m^2^. Approximately 30% of individuals aged 70 and above have a GFR below 60 mL/min/1.73 m^2^, (3) People with CKD who receive early diagnosis and treatment interventions and avoid renal replacement therapy, (4) People with CKD who receive early diagnosis and treatment interventions but experience unexpected renal injury events during the course of treatment, ultimately progressing to end-stage kidney disease, (5) People with CKD who progress to end-stage kidney disease because of delayed therapeutic interventions or lack of treatment. Abbreviations: CKD, chronic kidney disease; GFR, glomerular filtration rate; NSAIDs, non-steroidal anti-inflammatory drugs

If treatment interventions yield the same effect regardless of baseline GFR, intervening before GFR declines should delay progression to ESKD^10)^. A decline in GFR also increases the risk of cardiovascular disease onset. Thus, early treatment intervention is crucial because of the possibility of unexpected events leading to GFR decline, even where ESKD is not predicted to occur before the estimated age of death (Figure 4).

## Urinary albumin

Treatments to lower albuminuria are necessary because higher residual urinary albumin levels before and after treatment intervention are associated with a higher risk of future kidney events^11)^. The Chronic Renal Insufficiency Cohort (CRIC) study showed that even with CKD in the normal range of urinary albumin excretion (<30 mg/g), higher urinary albumin levels increased the rate of kidney event onset^12)^. Additionally, a meta-analysis of 15 observational cohort studies demonstrated that, compared with a urinary albumin level of <5 mg/g, levels of 5-10 mg/g or 10-30 mg/g were associated with a progressively increased risk of all-cause mortality and cardiovascular mortality^13)^.

**Figure 5:**
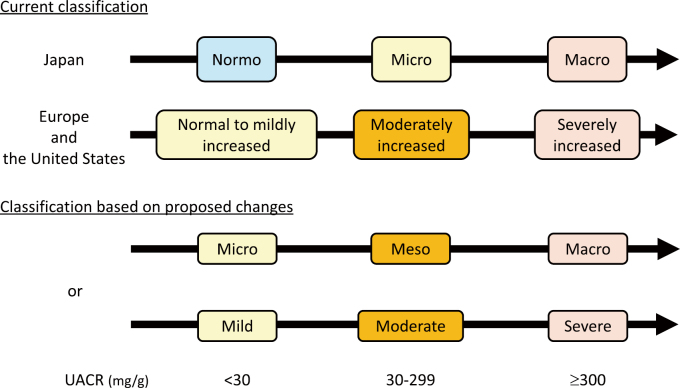
Classification of urine albumin excretion in people with diabetes Even within the normal range of urine albumin excretion (less than 30 mg/g), an increase in urine albumin is associated with an elevated risk of kidney and cardiovascular events. It may thus be beneficial to revise the current urinary albumin categories to help raise awareness and encourage treatment intervention. Abbreviations: UACR, urinary albumin-to-creatinine ratio

However, treatment interventions to lower urinary albumin are not standard when urinary albumin levels are in the normal range. This is likely because urinary albumin levels <30 mg/g are not considered a risk factor for cardiovascular disease or progression of CKD^14, 15)^. Additionally, no therapeutic agents have demonstrated renal protective effects for DKD with normal range of urinary albumin excretion (<30 mg/g). Furthermore, the term “normoalbuminuria” or “normal to mildly increased albuminuria” may have influenced physicians to believe that treatment was unnecessary. It may thus be beneficial to revise the current urinary albumin categories from normo (normal to mildly increased), micro (moderately increased), and macro (severely increased) to micro (mild), meso (moderate), and macro (severe) (Figure 5). Updating the current naming conventions could help raise awareness that urinary albumin levels are significant, even when the levels are below 30 mg/g and may encourage treatment intervention.

## Treatment goals for DKD

**Figure 6:**
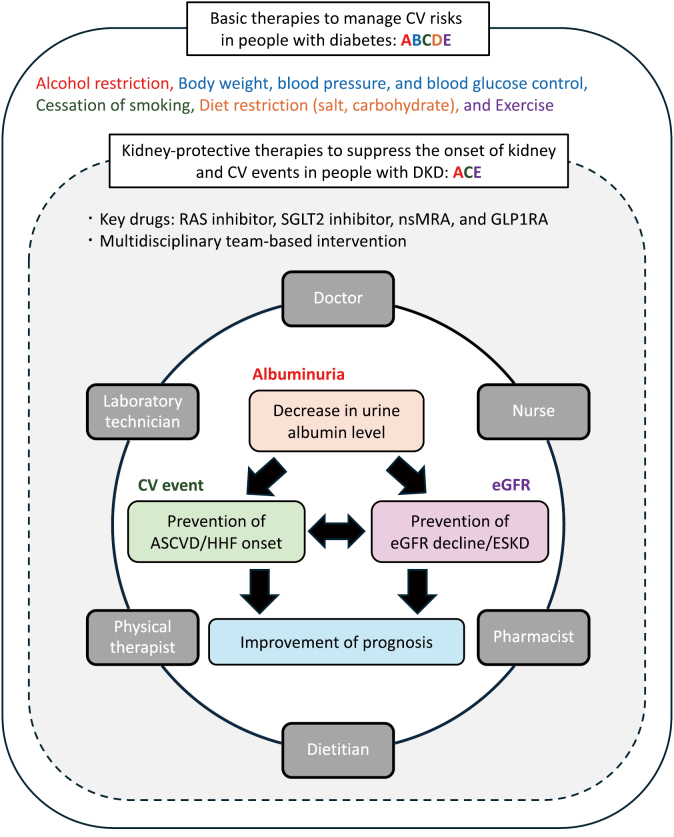
Treatment goals for diabetic kidney disease The overarching goal of treating people with diabetic kidney disease is to improve their lifetime prognosis. To achieve this, it is crucial to reduce albuminuria and prevent the onset of ESKD and CV events. Abbreviations: ASCVD, atherosclerotic cardiovascular disease; CV, cardiovascular; DKD, diabetic kidney disease; ESKD, end-stage kidney disease; eGFR, estimated glomerular filtration rate; GLP1RA, glucagon-like peptide-1 receptor agonist; HHF, hospitalization for heart failure; RAS, renin-angiotensin system; SGLT2, sodium-glucose co-transporter-2; nsMRA, non-steroidal mineralocorticoid receptor antagonist

The treatment goals for DKD are to reduce albuminuria, prevent the onset of atherosclerotic cardiovascular disease and heart failure, and suppress progression to ESKD, thereby improving lifetime prognosis. Additionally, slowing CKD progression helps prevent the worsening of heart failure, and vice versa. In other words, the health of the kidneys and heart are interrelated, with each organ significantly influencing the other. (Figure 6)^16)^. Unlike people of European origin, Japanese people have a higher risk of ESKD progression, but not all-cause mortality, even with GFR <30 mL/min/1.73 m^2^ ^17, 18)^. Therefore, it is essential to aim for prevention of ESKD progression even at the stage of kidney failure.

## Basic therapies for DKD

Dietary and exercise therapy should be implemented first along with lifestyle modifications such as appropriate weight management and smoking cessation, in addition to managing blood glucose, blood pressure, and lipids^19)^. Intervention by a multidisciplinary team can be expected to slow DKD progression^20)^. When kidney function is almost normal and urinary albumin levels are normal or low, strict intensive treatment for hyperglycemia, hypertension, and dyslipidemia can prevent the onset and progression of micro and macroalbuminuria, as shown in the J-DOIT3 trial^21)^.

RAS inhibitors are still an appropriate first-line therapy for hypertension with micro and macroalbuminuria. RAS inhibitors can reduce albuminuria and suppress kidney outcomes, including all-cause mortality in diabetes and macroalbuminuria^22)^. On the other hand, dual blockade therapies with angiotensin-converting enzyme (ACE) inhibitors and angiotensin II receptor blockers (ARBs), while showing albuminuria improvement, do not suppress cardiovascular event onset and increase the risk of hyperkalemia and acute kidney injury^23)^. Because the treatment goal for DKD is to prevent the onset of ESKD while avoiding side effects from medications, combination therapy with ACE inhibitors and ARBs is not recommended.

Strict blood glucose management aiming for glycated hemoglobin (HbA1c) below 6% in patients with a long history of diabetes or a history of cardiovascular disease can increase the risk of hypoglycemia, potentially raising all-cause and cardiovascular mortality risks^24)^. Therefore, blood glucose management should aim to keep HbA1c below 7% and to avoid hypoglycemia. There is insufficient evidence for the effect of strict blood glucose management in suppressing CKD progression in advanced DKD with macroalbuminuria.

## Novel DKD therapies after RAS Inhibitors

Recent large-scale clinical studies of people with diabetes have shown that SGLT2 inhibitors, nsMRAs, and GLP-1RAs can suppress kidney composite events (ESKD, 40%-57% decline in estimated GFR from baseline, kidney-related death)^5-7)^. Each of these trials tested the above therapies as an addition to standard treatment with RAS inhibitors, leading to significant improvements in the risk profile of DKD. Stratified analyses conducted within these trials showed renal protective effects, regardless of the presence or absence of RAS inhibitors. Additionally, in kidney outcome trials using SGLT2 inhibitors, no interaction on kidney outcomes was observed with or without the concomitant use of steroidal MRAs^25)^. Furthermore, in kidney outcome trials using nsMRAs, no interaction on kidney outcomes was observed with or without the concomitant use of SGLT2 inhibitors^26)^.

Actuarial analyses using data from large-scale clinical studies showed that, compared with RAS inhibitor monotherapy, the use of a combination of RAS inhibitor with one, two or all three of the following — a SGLT2 inhibitor, a nsMRA, or a GLP-1R inhibitor — progressively suppresses the incidence of hospitalization for heart failure and CKD events^27)^. Thus, novel DKD treatments may be expected to slow the progression of CKD, regardless of the presence or absence of RAS inhibitors. A highly anticipated trial is underway to examine the effects of combined therapies with nsMRAs and SGLT2 inhibitors, as well as each individually as monotherapy, on urinary albumin in people with type 2 diabetes and macroalbuminuria who are treated with RAS inhibitors^28)^.

## Conclusions

There is still insufficient evidence to provide granular criteria on who should be treated with these new therapeutic agents and in what order or combination they should be used. It is also known that these drugs do not necessarily provide renoprotection (albuminuria reduction) for all people with DKD^29, 30)^. Some people with DKD additionally have difficulty using these drugs because of side effects such as hyperkalemia, hypotension, and sarcopenia. Further investigation of the currently available treatments is needed. Additionally, the development of novel drugs is expected to continue to improve patient outcomes.

## Funding

No funding was received.

## Author contributions

TG conceived of the presented idea. YS, TK, KA, HH, KS, CK, EA, HS, SH, and MM collected data and provided content advice on the manuscript. All authors read and approved the final version of the manuscript.

## Conflicts of interest statement

The authors declare that there are no conflicts of interest.
